# Application of Guar Gum and its Derivatives as Green Binder/Separator for Advanced Lithium‐Ion Batteries

**DOI:** 10.1002/open.202100209

**Published:** 2022-02-01

**Authors:** Simran Kaur, Soumava Santra

**Affiliations:** ^1^ Department of Chemistry Lovely Professional University Phagwara Punjab 144411 India

**Keywords:** application, green binder, guar gum, lithium-ion battery, separator

## Abstract

Since their first commercialization in the 1990s,lithium‐ion batteries (LIBs) have become an indispensible part of our everyday life in particular for portable electronic devices. LIBs have been considered as the most promising sustainable high energy density storage device. In recent years, there is a strong demand of LIBs for hybrid electric and electric vehicles to lower carbon footprint and mitigate climate change. However, LIBs have several issues, for example, high cost and safety issues such as over discharge, intolerance to overcharge, high temperature operation etc. To address these issues several new types of electrodes are being studied. Traditional binder PVDF is costly, difficult to recyle, undergoes side reactions at high temperature and cannot stabilize high energy density electrodes. To overcome these challenges, diiferent binders have been introduced with these electrodes. This minireview is focused on the application of guar gum as a binder for different electrodes and separator. The electrochemical performance of electrodes with guar gum has been compared with other binders.

## Graphical abstract

**Bind naturally to perform**: Adoption of suitable binder imparts remarkable effect on the electrochemical performance of electrodes in LIBs. Binders can alleviate several issues faced by different types of electrodes and separators. In this review, electrochemical performance of different electrodes for LIBs and separator with an abundant, low‐cost, renewable, green binder guar gum as well as its derivatives have been discussed and compared with other binders, for example, PVDF.

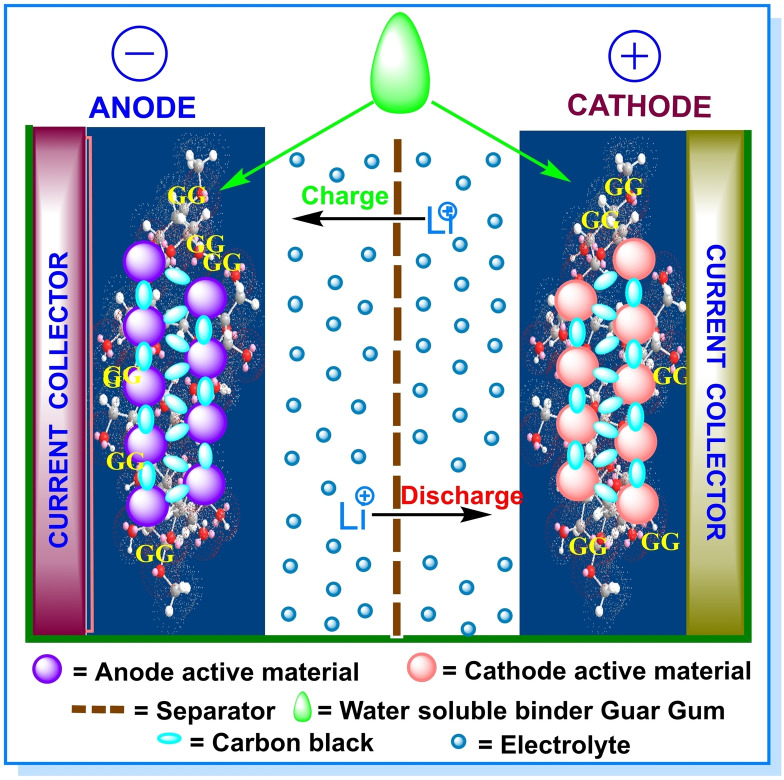

## 1 Introduction

Over the past few centuries, human civilization has primarily relied upon fossil fuels to generate energy for transportation, industry and other basic needs.^[1]^ Continuous use of fossil fuels may potentially make us vulnerable in future as there may be severe shortage of fossil fuels. In addition, use of fossil fuels for energy generation, typically causes environmental pollution which leads to many health issues.[^2^, ^3^] More importantly, climate change has ocuured due to the unfettered utilization of fossil fuels.[^4^, ^5^] Therefore, there is an urgent need for alternative energy sources which could be sustainable and environmentally friendly.

**Figure 1:**
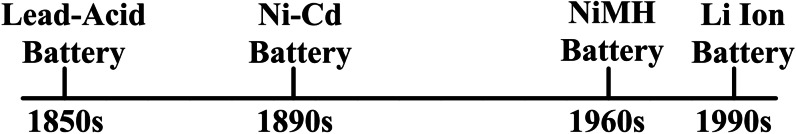
Timeline of rechargeable battery developments.

Consequently, solar, wind, geothermal, hydro, tidal and marine energy research have progressed to harvest energy from these renewable sources.^[6]^ However, one major issue was the storage of harvested energy from these sustainable methods for the round‐the‐clock and uninterrupted supply of energy.^[7]^ A rechargable battery is the most suitable option for this purpose. As a result, from the lead‐acid battery (LAB) in the 1850s to the evolution of lithium‐ion battery (LIB) in the 1990s (Figure 1) has occured to address several issues like cost, tolerance of temperature, rate capability and cycle life.^[8]^

LIBs as secondary batteries are in great demand for their extensive application as a sustainable/green power source in electronic gadgets in our daily life. Astonishingly, they are poised to transform modern transportation system with their use in hybrid electric vehicles (HEVs), electric vehicles (EVs), for example, cars, buses, bikes and smart power grids.[^9^, ^10^, ^11^] This is primarily due to the small size, light weight, high energy density, high power density and long durability or lifespan. In addition, LIBs exhibit high open circuit voltage, almost zero‐memory effect and low self‐discharge properties.[^12^, ^13^, ^14^] Inspite of these, there are some drawbacks such as (i) increasing internal resistance with aging/cycling, (ii) moderate cycle life, (iii) associated overheating/overcharged safety concerns.^[15]^ These issues have fueled further research in the present era of LIBs and, much of the current impetus of research on LIBs is primarily because of the strong demand of LIBs for HEVs, EVs which can be charged fast, driven to longer distance and, also reduce greenhouse gases.[^16^, ^17^] In LIBs, graphite, silicon, ZnCo_2_O_4_, Li_4_Ti_5_O_12_ have been employed as anode; while sulfur, lithiated transition metal oxides have been used as cathode. These electrodes offer different specific capacity and energy density.[^18^, ^19^, ^20^]

**Figure 2:**
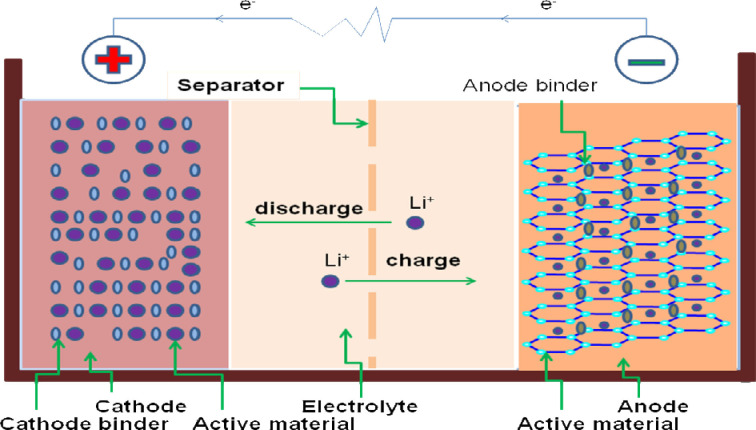
General structure of a lithium‐ion battery.

In typical LIBs, cathode and anode are separated by a separator and electrolyte is present in between them (Figure 2). The cathode is composed of lithiated metal oxides (e. g. LiMO_2_, M=Co, Ni, Mn etc.) and, the anode is of graphite or carbon.[^21^, ^22^, ^23^] It works via the well known intercalation chemistry of host and guest ion/molecule.^[24]^ The performance of LIBs largely depends upon the active materials of cathode and anode. The cathode is often referrred to as the “achillies heel” of LIBs and consequently, layered oxides, spinel oxides, polyanion oxides, sulfur are being deveoped to address several issues. As an anode material, graphite has low theoretical capacity (372 mA h g^−1^) and poor rate capability.^[25]^ This limits the energy density of LIBs and, raises the safety issues.[^18^, ^19^, ^20^] Several studies have been done in order to increase the specific capacity as well as energy density of LIBs. In those studies, graphite electrode has been replaced with other electrodes for example, silicon, ZnCo_2_O_4_. Li‐ternary ZnCo_2_O_4_ (ZCO) battery offers theoretical capacity of ∼900 mA h g^−1^, Li−Si offers capacity 4200 mA h g^−1^. Substitution of graphite with other electrodes is a challenging issue. Electrodes like silicon changes their volume on lithiation and de‐lithiation processes, even further pulverization is another challenging issue.[^26^, ^27^, ^28^, ^29^, ^30^, ^31^, ^32^, ^33^, ^34^, ^35^, ^36^] To overcome these challenges, several strategies have been investigated for example novel architechture of electrodes, functional separator, nano‐scale active materials. Furthermore, synthetic and natural compound‐based binders have also been incorporated with the electrodes.[^37^, ^38^, ^39^, ^40^, ^41^, ^42^, ^43^, ^44^, ^45^, ^46^, ^47^, ^48^, ^49^, ^50^, ^51^, ^52^, ^53^, ^54^, ^55^]

The binders are essential component for the fabrication of LIBs. The passive binders generally improve inter‐connectivity within the electrodes (active material, current collector, conductivity enhancer) and, also facilitates ionic conductivity. Thus, the binders typically alleviate irreversible capacity loss, stabilize the electrode structure, increase the charging‐discharging cycle, improve safety and, thus they make the battery more like a super capacitor which can have many rapid charge/discharge cycle and provide quick energy boost.[^56^, ^57^, ^58^, ^59^, ^60^, ^61^] Binders are broadly classified into two categories: (i) organic solvent soluble binders and, (ii) water soluble binders. Advanced binders are further classified into strong affinity, 3D network, conducting, redox‐active and biopolymer binders.

**Figure 3:**
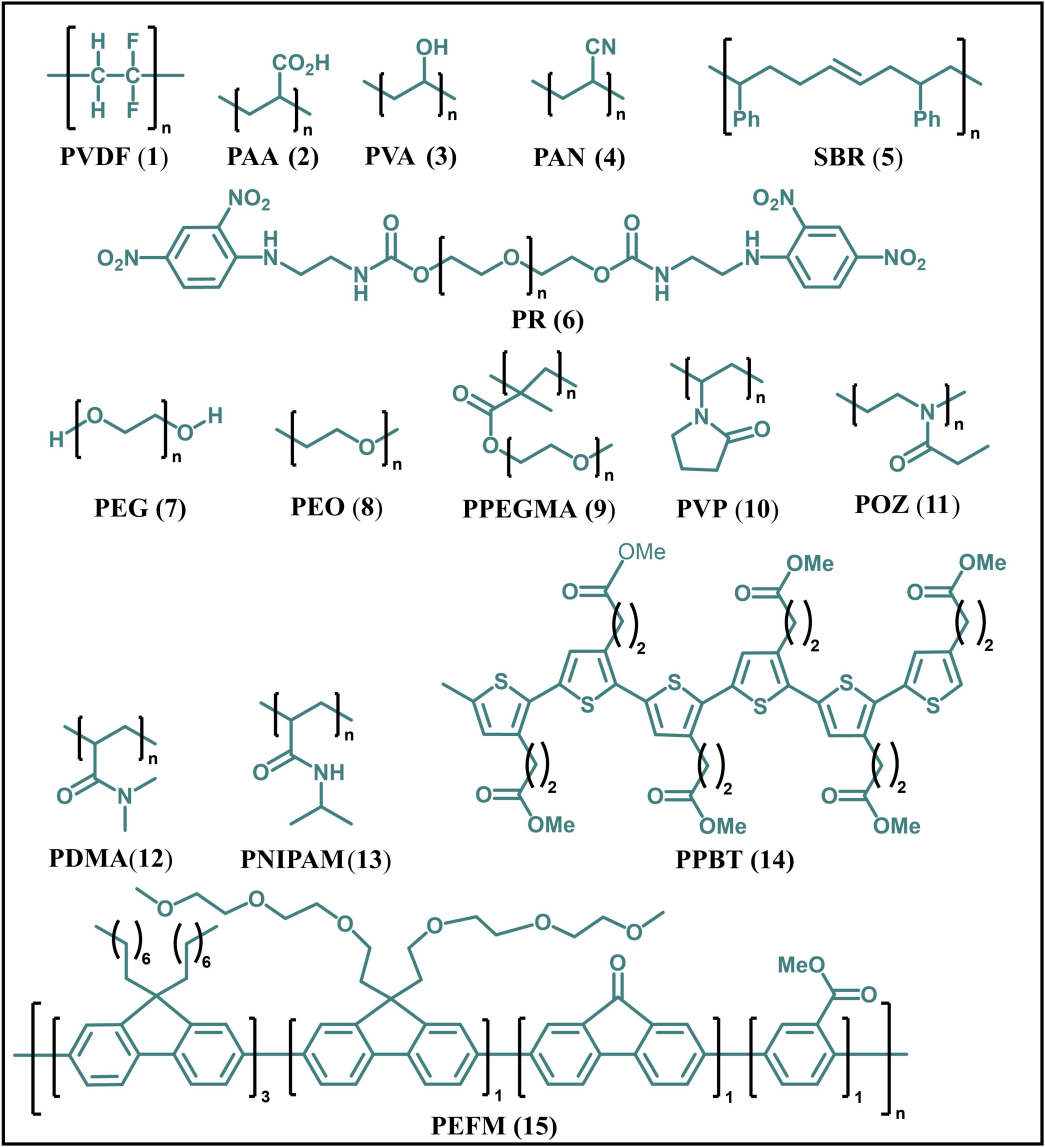
Examples of synthetic binders used for LIBs.

Synthetic polymeric binders, for example, polyvinylidene fluoride (PVDF, **1**), polyacrylic acid (PAA, **2**), polyvinyl alcohol (PVA, **3**), polyacrylonitrile (PAN, **4**), styrene butadiene rubber (SBR, **5**), polyrotaxanes (PR, **6**), polyethylene glycol (PEG, **7**), polyethylene oxide (PEO, **8**), poly(polyethylene) glycol methyl ether methacrylate) (PPEGMA, **9**), polyvinyl pyrrolidone (PVP, **10**), poly(2‐ethyl‐oxazoline) (POZ, **11**), poly(*N*,*N*‐dimethylacrylamide) (PDMA, **12**), poly(*N*‐isopropylacrylamide) (PNIPAM, **13**), poly[3‐(potassium‐4‐butanoate) thiophene‐polyethylene glycol (PPBT, **14**), poly(2,7,9,9‐dioctylfluorene‐co‐2,7,9,9‐(di(oxy‐2,5,8‐trioxadecane))fluorene‐co‐2,7‐fluorenone‐co‐2,5,–1‐methoxybenzoate ester (PEFM, **15**) and, MXene have been investigated for LIBs (Figure 3).

Traditional binders, for example PVDF, a linear homopolymer, are soluble in organic solvents and, have been widely used in commercial LIBs because of their good adhesion as well as electrochemical stability on commercial electrodes. However, PVDF is costly (19–25 $/kg) and, requires *N*‐methyl pyrrolidone (NMP) solvent, which is a volatile organic compound and, classified toxic substance. In addition, it melts at elevated temperature (>165 °C) and, recovery of PVDF is a costly process. Above 165 °C, PVDF undergoes side reaction with lithiated graphite or lithium metal to form resistive side‐products. More importantly, PVDF cannot stabilize the structure of high energy density electrode materials due to its non‐polar nature.

**Figure 4:**
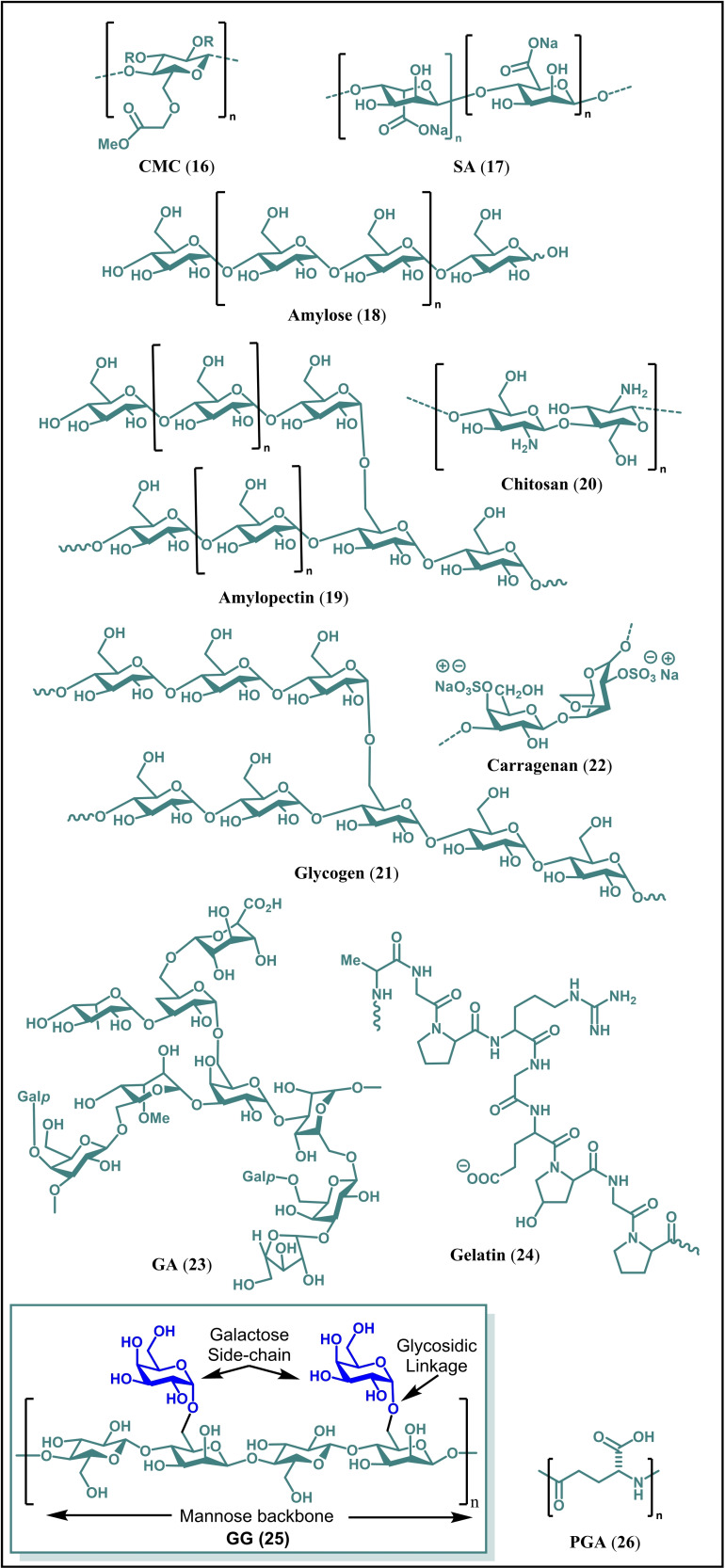
Examples of natural binders used for LIBs.

In this regard, natural binders having coordinating groups and 2D/3D framework may potentially overcome aforementioned drawbacks of PVDF. Consequently, several natural biopolymers, for example, carboxymethyl cellulose (CMC, **16**), sodium alginate (SA, **17**), amylose (**18**), amylopectin (**19**), chitosan (**20**), glycogen (**21**), carragenan (**22**), gum arabic (GA, **23**), gelatin (**24**), guar gum (GG, **25**), poly‐γ‐glutamate (**26**), lignin, pectin, potato starch, wheat starch, xantham gum (XG), β‐cyclodextrin polymer and soy protein in their native or crosslinked form have been investigated as binders for different LIBs, for example Li−Si batteries to overcome aforementioned challenges (Figure 4).[^38^, ^53^, ^56^, ^57^, ^65^, ^66^]

GG (**25**) is a water‐soluble, non‐toxic, abundant, renewable, natural heteropolysaccharide of the galactomannan family. It is comprised of d‐mannose straight chain branched with d‐galactose through (1–4)‐β glycoside linkages. GG is also a low‐cost (1–3 $/kg) non‐ionic, biodegradable and eco‐friendly polysaccharide.[^67^, ^68^, ^69^] GG and its derivatives have found wide practical applications due to their unique properties.[^70^, ^71^, ^72^, ^73^, ^74^, ^75^] In lieu of these applications, several studies have also been done using GG and its derivatives as a strong affinity biopolymeric binding agent for LIBs to address aforementioned issues. For example, GG has been used as a binder in Li−Si, Li‐Sulfur and Li‐titanate batteries.[^76^, ^77^, ^78^] In addition, it has also been used as binder in electrochemical double layer capacitors and, separator for LIBs.[^79^, ^80^] Herein, we review the recent progress on the application of GG and its derivatives as a binder/separator for the next generation LIBs which are in the development process.

## 2 GG as a Binder for LIBs

Being an eco‐friendly and non‐toxic heterobiopolymer, GG has found great importance as an advanced binder in LIBs. Various studies have shown that modification of hydroxyl groups of GG can modulate its physical properties. Thus, the feasibility of GG as well as its chemically modified derivatives have been investigated as binders for LIBs. These applications have been categorized according to the type of electrodes in which GG was employed as a binder.

### 2.1 Silicon Based Anode

As an alternative to traditional graphite anode, silicon (Si) has got immense attention in recent years for the development of high energy density LIBs. This is primarily because Si anode can have a specific capacity of >3500 mA h g^−1^. Additionally, being the second most abundant element, Si anode could be comparatively much cheaper and eco‐friendly. Furthermore, Si anode has low operating voltage −0.2–0.4 V than Li/Li^+^ and, the silanol groups (SiOHs) on the surface of Si anode can interact with the binding materials.[^37^, ^81^, ^82^] However, large volume change of Si anode occurs (∼300–400 %) upon lithiation (Si→Li_4.4_Si) and delithiation processes which destabilizes the electrode structure to a great extent.[^37^, ^83^, ^84^] Even composite and nano‐structured Si material get fractured during cycling because of intrinsic volume change and, also due to continuous decomposition of electrolyte on their surface. Liu et al. reported that GG can be used as a robust binder for Si nanoparticle (SiNP) anode.^[78]^ The authors showed that GG has a relatively high viscosity (5600 mPa s) and exhibits high ionic‐conductivity due to the presence of numerous polar hydroxyl groups.^[37]^ These properties make GG a relatively harder binding agent. The authors mentioned that GG had improved the electrochemical performance of SiNP anode and, provided new binding design to the SiNP anode of LIBs. Interaction between the hydroxyl groups of GG and SiNP anode increased the cycle performance of LIBs.

**Figure 5:**
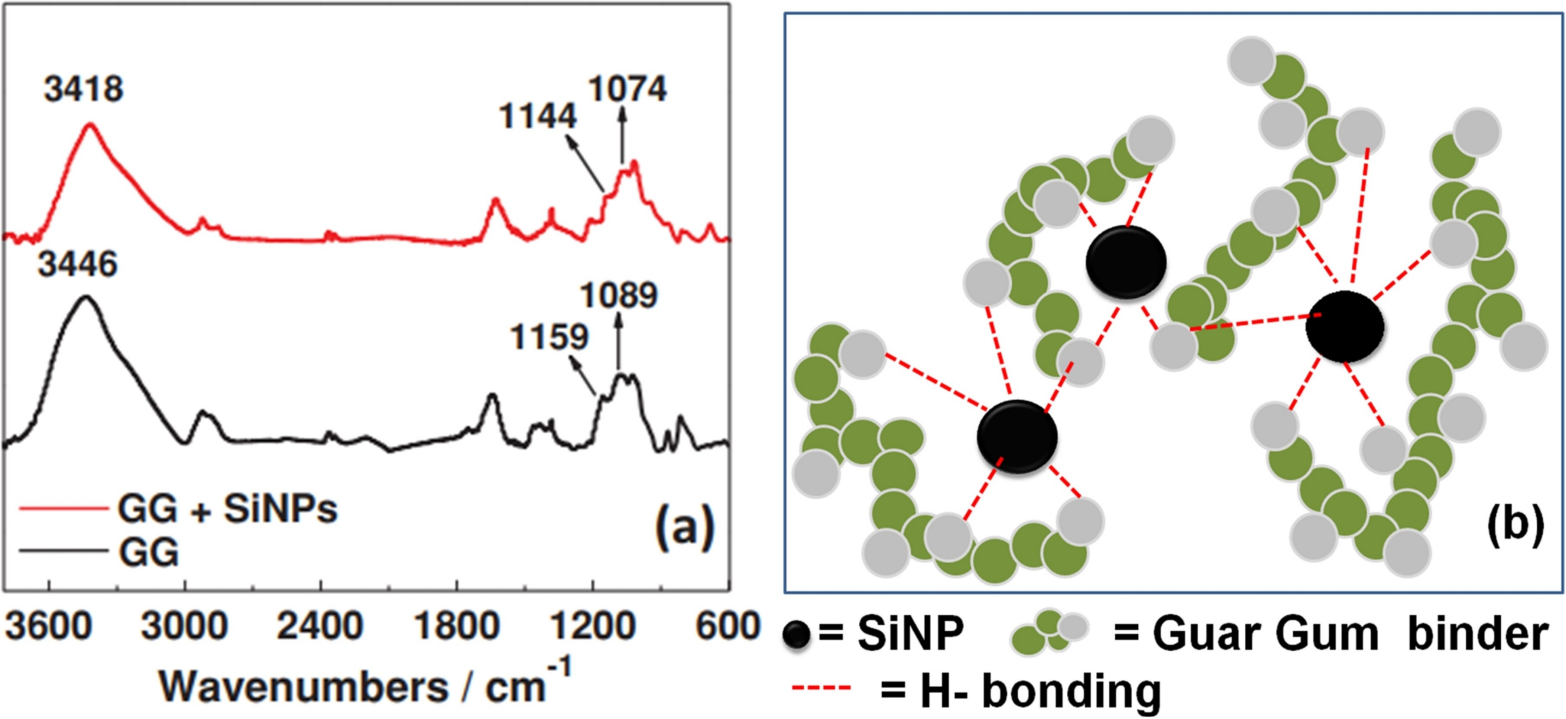
(a) FTIR spectra of GG and GG+SiNPs. Reproduced from Ref. [78]. Copyright 2015, Wiley VCH. (b) Schematic representation of H‐bonding between GG and SiNPs.

Fourier‐Transform Infrared (FTIR) spectroscopy revealed that there are interactions between the OH groups of GG and the Si‐anode. Pure GG showed three characteristic peaks – a broad peak around 3446 cm^−1^ which corresponds to the O−H stretching, C−OH stretching peak at 1159 cm^−1^ and CH_2_−OH stretching peak at 1089 cm^−1^.[^85^, ^86^] When pure GG was bound to SiNPs, the stretching peaks for O−H, C−OH and CH_2_−OH shifted to 3418, 1144, 1074 cm^−1^ respectively (Figure 5a). Shifting of the stretching frequencies of these peaks to lower wavelengths indicated that interaction between GG and SiNPs has occured.[^81^, ^82^, ^87^]

Analysis of the binding strength of GG with SiNPs was done by thermogravimetric analysis (TGA). It was found that 32 % of GG remained on SiNPs after washing. This confirmed that GG was bound strongly to the SiNPs through hydrogen bonding (H‐bonding) due to the presence of numerous polar hydroxyl group binding sites as depicted in Figure 5b.

**Figure 6:**
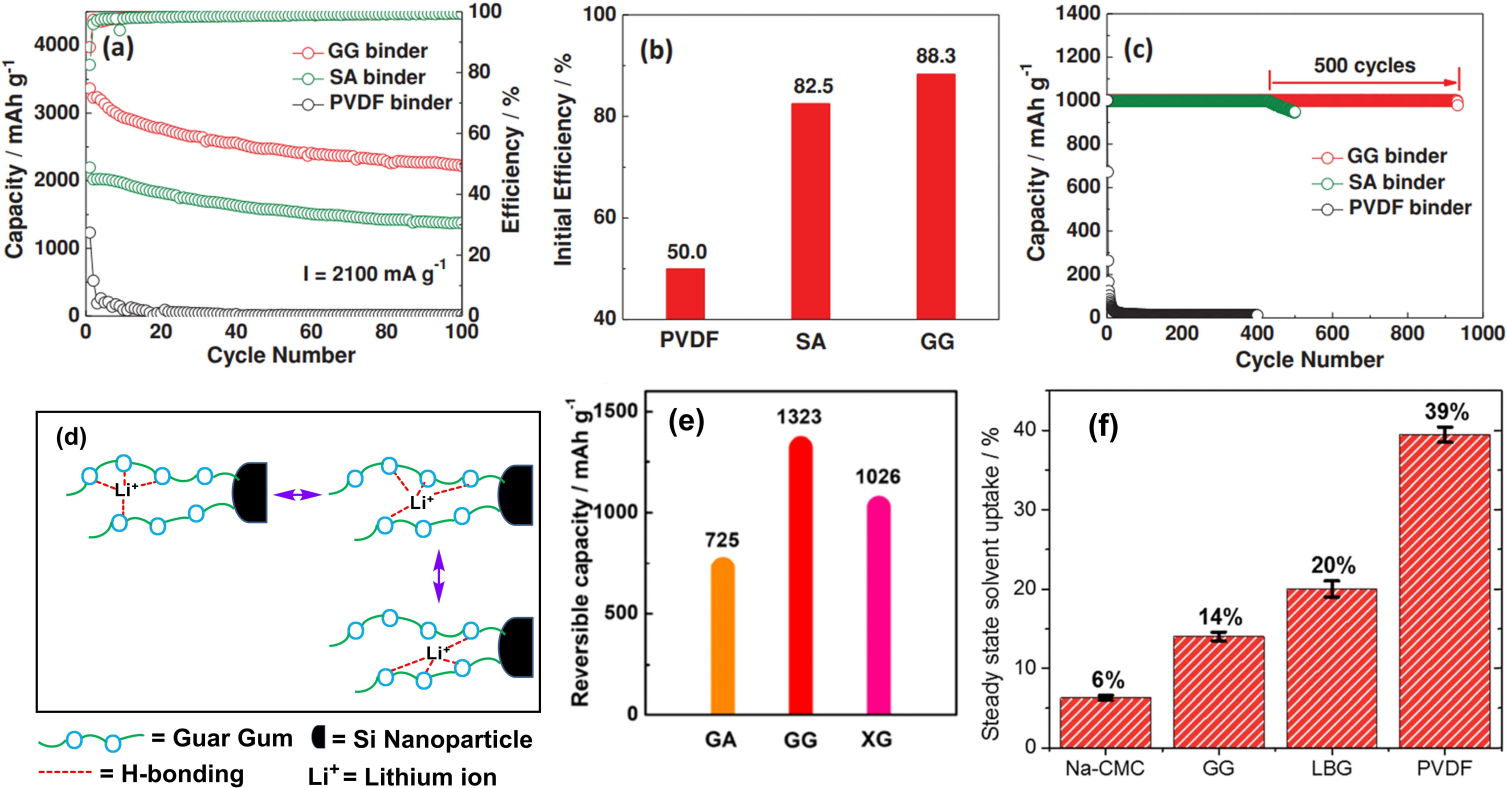
Electrochemical performance of SiNP anodes with different binders. a) Cycle performance at 2100 mA g^−1^ between 0.01 and 1.2 V; b) Initial efficiency at 2100 mA g^−1^; c) Cycle performance with limited discharge capacity of 1000 mA h g^−1^at 1000 mA g^−1^. Reproduced from Ref. [78]. Copyright 2015, Wiley VCH. d) Schematic illustration of Li‐ion hopping in the presence of GG, (e) Retention of capacity of GA, GG, GA after 100 cycles. Reproduced from Ref. [88]. Copyright 2021, Springer. (f) Volume change of Na‐CMC, GG, LBG, PVDF films at steady state after solvent uptake (in 1 M LiPF_6_). Reproduced from Ref. [89]. Copyright 2015, Royal Society of Chemistry.

When the initial efficiency of SiNP anode with different binders was compared, it was found that GG, SA and PVDF‐bound SiNP anode delivered 3364 mA h g^−1^, 2195 mAh g^−1^, 1232 mA h g^−1^ discharge capacity respectively at a current density of 2100 mA h g^−1^. More importantly, after 100 charge‐discharge cycle, the SiNP anode with GG, SA and PDVF retained 2222 mA h g^−1^, 2195 mA h g^−1^, 1377 mA h g^−1^ of discharge capacity respectively (Figure 6a). Among these binders, GG showed highest initial Columbic efficiency of 88.3 % whereas SA and PVDF delivered 82.5 % and 50.0 % respectively (Figure 6b). When the initial discharge capacity was limited to 1000 mA h g^−1^, GG‐bound SiNP anode was able to deliver same capacity upto 1000 cycle, whereas for SA it started to decrease after 400 cycle (Figure 6c). In between 0.1–3.0 V, pure binders (GG and SA) were found to be electrochemically inactive. This was evident from the oxidation‐reduction current which was relatively weak in comparison to SiNP electrode. The electrode structure was highly stablized because of the formation of network of H‐bonding due to the presence of large number of hydroxyl groups. This in turn reduced the number of isolated Si and thereby, delivered initial high capacity. In partcular, the lone pairs on the heteroatom oxygen formed complex with the lithium ion and, then gets dissociated due to thermal motion which arose from the generated heat. The lithium ion then complexed with new sites (O atoms) and, thus hopping of lithium ion occured through the aid of GG (Figure 6d).

When the capacity retention of GA, GG and xanthum gum (XG) based SiNP electrodes were compared at 400 mA g^−1^, GG again delivered better capacity (1323 mA h g^−1^) after 100 cycles (Figure 6e).^[88]^ The binders also swell to some extent through electrolyte uptake and, this may facilitate the transport of Li^+^ ion. Submerged films of Na‐CMC, GG, locust bean gum (LBG), PVDF in electrolyte, were found to be swelled as their masses were increased by 6 %, 14 %, 20 % and 39 % respectively (Figure 6f).^[89]^ As a result, SiNP electrodes with GG binder can perform better since more Li^+^ ion can be inserted or extracted. Additionally, the swelling also helped to minimize britlleness tendency of GG upon lithiation. Furthermore, extensive H‐bonding network with SiNP anode, may mediate self‐healing process of GG binder if any minor fracture takes place during charge/discharge cycles.

### 2.2 Lithium‐Sulfur Battery

Owing to the natural abundance, eco‐friendliness, non‐toxic nature and, five‐times higher theoretical energy density (2600 W h kg^−1^) than conventional LIBs (∼500 W h kg^−1^), Li‐Sulfur batteries (LISBs) have gained wide attention in recent years as a cheaper energy storage device. In LISBs, the cathode and anode are composed of elemental sulfur (S_8_) and lithium respectively. LISBs may find major applications in smart grids and electrical vehicles.[^90^, ^91^] However, rechargeable LISBs face several critical issues those need to be addressed – (a) during charging‐discharging processes, LISBs face nearly 76 % expansion or shrinkage of sulfur electrode, (b) dissolution of in situ formed long chain polysulfide from the cathode active material, (c) shuttle reaction at the anode, (d) insulating nature of sulfur and, (e) decrease of Coulombic efficiency.[^92^, ^93^] For complete drying, traditional binder PVDF is required to be heated at 120 °C. Active material is being lost in this process because sulfur gets vaporized at this temperature and, also leading to environmental issues.^[94]^ As a result, the LISBs have short life‐span. Although, nano‐structured carbon additive was able to improve cycle life as well as electrochemical performance of LISBs, however, these sophisticated methods are costly which limits their large‐scale manufacturing.[^95^, ^96^]

To overcome these problems, in particular the “shuttle effect”, advanced and rationally designed binders are required because binders play critical roles by holding the active material on the electrode via active binding, enhancing electrical contact between the conductive carbon and active material and, linking current collector with the active material. Binders can also limit the active material's dissolution in electrolyte.[^98^, ^99^] According to the ab initio calculations of Li_2_S‐binder and Li_2_‐S^.^‐binder under the density function theory (DFT) by She et al., binders having electron‐rich groups bearing oxygen, nitrogen or halogen atoms can bind to the lithium ion which are part of the polysulfides. This is primarily due to the presence of lone pair of electrons on the hetero atoms of the electron‐rich groups of the binders.^[99]^ It was found that compounds having carbonyl groups have shown strongest binding energy (1.20–1.26 eV) with Li_2_‐S^.^ species (Table 1). Configuration in which lithium ion was directly coordinated to the oxygen of C=O, found to be most stable.

**Table 1:** Calculated binding energy of selected functionalities with Li_2_−S^.^ species from ab initio calculation.

Entry	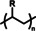	Compound Class	Binding Energy with Li−S^.^ [eV]
1	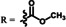	Ester	1.26
2	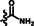	Amide	1.23
3	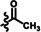	Ketone	1.2
4	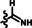	Imine	1.02
5	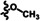	Ether	1.01
6	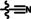	Nitrile	0.77
7	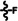	Fluoroalkanes	0.62
8	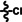	Chloroalkanes	0.46
9	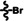	Bromoalkanes	0.42
10	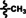	Alkane	0.30

**Figure 7:**
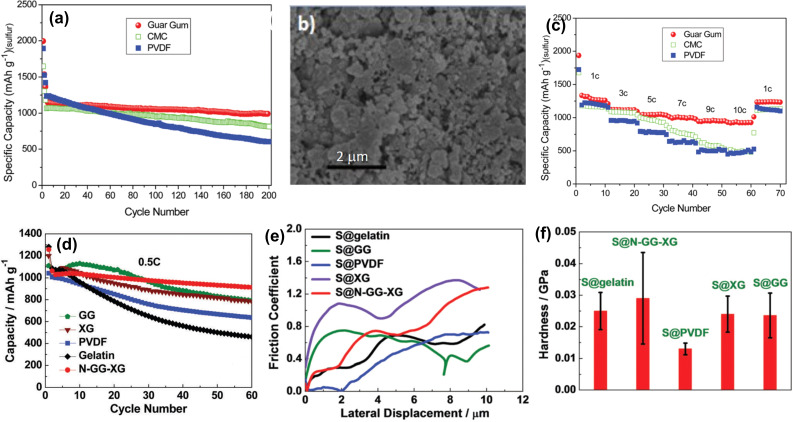
(a) Comparison of discharge capacity of S@pPAN electrode with GG, CMC and PVDF binder at C/5. (b) SEM image of S@pPAN electrode with GG after 100 cycles. (c) Rate capability of S@pPAN electrode with GG, CMC and PVDF binders at 0.2 C. Reproduced from Ref. [107], Copyright 2016, Royal Society of Chemistry. (d) Comparison of cyclic performance of S@GG, S@XG, S@PVDF, S@Gelatin, S@N‐GG‐XG cathodes at 0.5 C. (e) Friction coefficients of S@GG, S@XG, S@PVDF, S@Gelatin, S@N‐GG‐XG cathodes (nanoscratch test). (f) Hardness of S@GG, S@XG, S@PVDF, S@Gelatin, S@N‐GG‐XG cathodes (nanoindentation test). Reproduced from Ref. [108], Copyright 2017, Royal Society of Chemistry.

In addition, electrode materials as well as electrolytes have great impact on the cycle performance of battery and, consequently, various composites of sulfur have been prepared.[^100^, ^101^] For example, Wang et al. reported the synthesis of cathode material (S@pPAN) obtained by the reaction between elemental sulfur and polyacrylonitrile (PAN).[^102^, ^103^, ^104^] Utilization of GG as a binder for LISBs has been reported by Qinyu Li et al.^[107]^ According to the authors, the S@pPAN cathode with GG binder delivered a discharge capacity of 1469.2 mA h g^−1^ which was higher compared to CMC binder. When the cycle performance with GG binder after 50 cycles was compared, the S@pPAN cathode was able to maintain a discharge capacity of 1375 mA h g^−1^, however with PVDF it was 958 mA h g^−1^. This clearly indicated that GG was superior than CMC and PDVF as shown in Figure 7a.

Scanning electron microscope (SEM) images of S@pPAN electrode with GG binder after 100 cycles showed no visible fracture or pulverization (Figure 7b). In comparison to PVDF binder, lower interfacial impedance was also observed with S@pPAN/GG electrode. Therefore, the S@pPAN/GG electrode had maintained its structural integrity after 100 cycles while crack was observed in the corresponding S@pPAN/PVDF electrode. This was further confirmed by CV experiments. In terms of rate capability, S@pPAN/GG electrode exhibited 1280, 1050, 1000 and 930 mA h g^−1^ of capacities at 1 C, 5 C, 7 C and 10 C respectively. On the other hand, corresponding S@pPAN/CMC and S@pPAN/PVDF electrodes exhibted gradual decrease in rate capabilities, for example, at 9 C, ∼530 and ∼500 mA h g^−1^ respectively (Figure 7c).

A 3D network binder comprised of GG and XG was developed by Liu et al.^[108]^ They have leveraged the intermolecular H‐bonding between GG and XG for their robust 3D biopolymer (N‐GG‐XG).[^109^, ^110^] The interaction between GG and XG took place at the smooth region of XG where galactopyranose units were absent. Shifting of O−H,C=O peaks in the FTIR spectrum and change of peak intensities in the X‐Ray Diffraction (XRD) confirmed the intermolecular interaction.^[37]^

The S@N‐GG‐XG electrode exhibited two plateau in CV near 2.1 V and 2.3 V at which insoluble short‐chain (Li_2_S_2_/Li_2_S) and soluble polysufides (Li_2_S_x_) having long chain (4≤x ≤8) typically forms respectively in conventional LISBs. This has validated the suitability of N‐GG‐XG as a binder for LISBs. In terms of cyclic performance, S@N‐GG‐XG was superior than S@PVDF, S@gelatin and even, slightly better than GG as well as XG alone (Figure 7d). After 60 cycles, the S@N‐GG‐XG was able to maintain a discharge capacity of 913 mA h g^−1^ which was ∼2‐fold higher than S@gelatin and ∼3.3‐fold higher than S@PVDF. Even after 150 cycles, the S@N‐GG‐XG exhibited a capacity of 724 mA h g^−1^ and, also exhibited an excellent rate capability at 5 C. Moreover, the discharge capacity of the S@N‐GG‐XG with a high sulfur loading (11.9 mg cm^−2^) was 733 mA h g^−1^. This could be primarily due to the 3D network and presence of numerous oxygen atoms.

The authors also evaluated mechanical properties of S@N‐GG‐XG, S@GG, S@XG, S@PVDF and S@gelatin using scanning probe microscopy (SPM). In the nanoscratch test with SPM, the S@N‐GG‐XG showed a smooth scratch track, while others exhibited irregular patterns (Figure 7e). This confirmed that the N‐GG‐XG binder has much higher ability to tolerate stress induced by volume change. Nanoindentation test under a force of 500 μN showed that the N‐GG‐XG binder has high friction coefficient and, therefore, exhibited highest hardness property because it possessed high adhesive force (Figure 7f). Consequently, the S@N‐GG‐XG electrode exhibited better cyclic performance than the electrode with other binders.

The authors had employed super P carbon (SPC) as a conductive matrix which typically absorbs low amount of sulfur because the surface area of SPC is low and, less porous. In addition, LiNO_3_ (1.5 wt%) was employed to reduce the “shuttle effect”. However, the “shuttle effect” still has occurred when there was low N‐GG‐XG content (10 %) and high sulfur loading (11.9 mg cm^−2^). Surprisingly, the S@N‐GG‐XG electrode exhibited normal charge‐discharge profile when the sulfur loading was even 19.8 mg cm^−2^. The prepared LISB battery was able to power up a stop‐watch and light‐emitting diode (LED) which further confirmed the charge storage capability and high operating voltage. It has been suggested that employment of more LiNO_3_ in combination with conductive matrix having porous carbon, might further improve the cyclic performance of LISBs.

### 2.3 Lithium‐Titanate Based Anode

Transition metal containing anode, for example, spinel structured lithium titanate or lithium titanium oxide (Li_4_Ti_5_O_12_) or LTO have also been employed in LIBs which was successfully commercialized recently. In LTO, CMC binder was reported to exhibit better electrochemical performance than PVDF.[^111^, ^112^] It was observed that the adhesion of CMC having high molecular weight (MW) and low degree of substitution (DS) was higher than CMC having low MW and high DS. Lee et al. carried out a comparative study to determine the efficacy of GG, Tara Gum (TG) and CMC as binder for LTO.^[113]^

**Figure 8:**
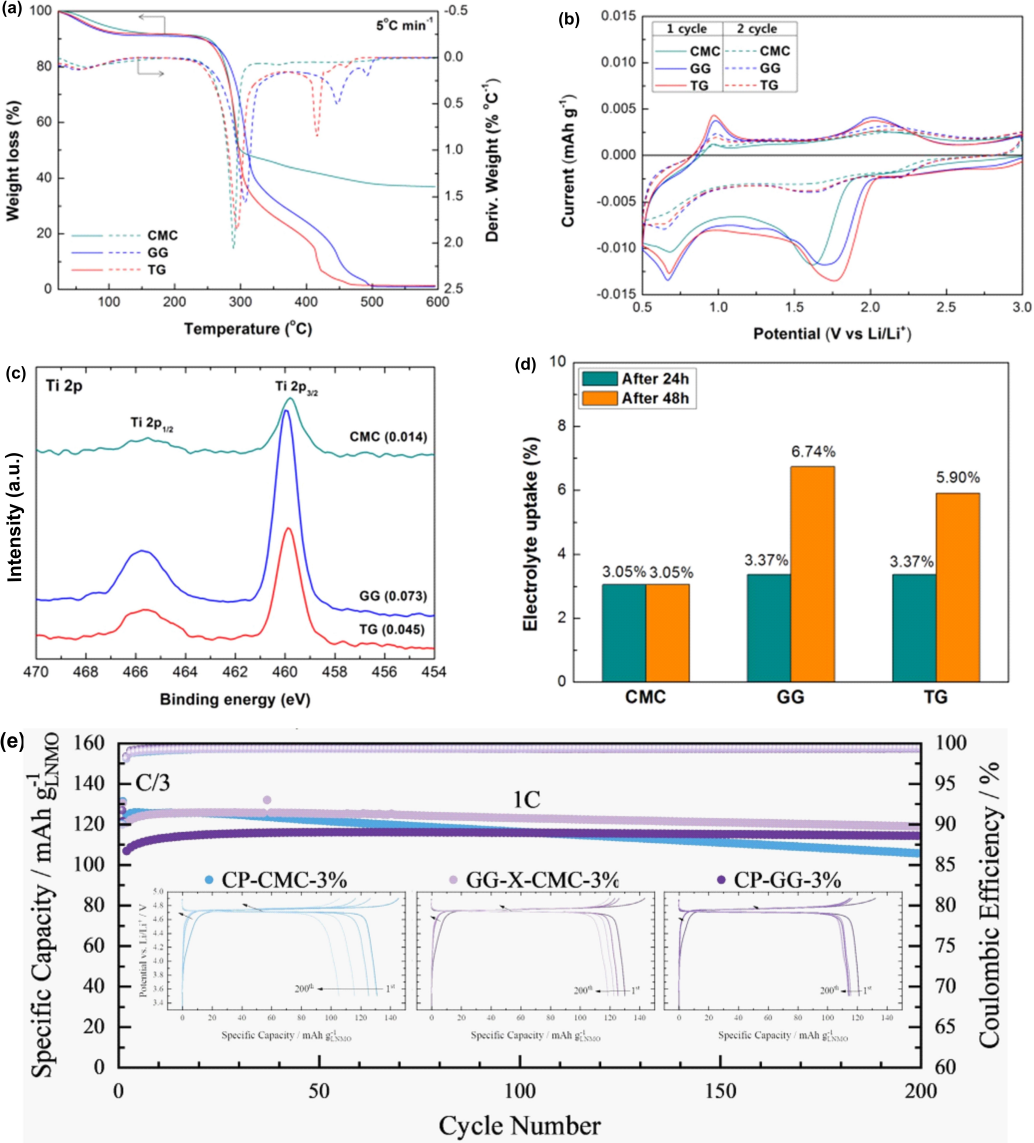
(a) TGA curves of CMC, GG and TG. (b) CV curves of CMC, GG and TG. (c) Ti 2p spectra of LTO with CMC, GG and TG. (d) Uptake of electrolyte by CMC, GG and TG after 2 days in solvent‐free electrolyte. Reproduced from Ref. [113]. Copyright 2014, The Electrochemical Society. (e) Constant current cycling and selected potential profiles of LNMO@CP‐CMC‐3 %, LNMO@GG−X‐CMC‐3 % and LNMO@CP‐GG‐3 % after 200 cycles. Reproduced from Ref. [115]. Copyright 2020, Wiley VCH.

The composite electrodes associated with binders are subjected to high temperature for their drying process and electrolytes during working of batteries. Therefore, the electrochemical and thermal stability of binder films are very crucial. Stability of GG, TG and CMC binder films were analyzed by TGA as shown in Figure 8a.

The TGA curve showed that slight weight loss has occured in all binders below 100 °C. This loss of weight has been attributed to the desorption of the water molecules which were adsorbed on the binders surface due to the presence of numerous hydroxyl groups. Compared to GG and TG, CMC exhibted higher water absorbing capacity due to the presence of *cis*‐hydroxyl groups.^[106]^ In comparison to CMC, the backbone polymer chain of GG and TG tend to degrade at higher temperature (∼250 °C). However, due to the presence of strong H‐bonding between adjacent chains, CMC was found to be resistant to degradation at temperature higher than 300 °C. The degradation of the polymeric backbones of CMC, GG and TG has been attributed to the elimination of CO_2_ because C−O−C bonds get cleaved. Thus, GG and TG exhibited sufficient thermal stability to be used as binders.

In the CV cycles, under cathodic scan, only two redox peaks at 0.68 V and 1.78 V and, under anodic scan at 0.97 V and 2.0 V were observed and, they were reversible in nature (Figure 8b). Therefore, like CMC, GG and TG were also electrochemically stable enough in the operating voltage of LTO (0.3–5.0 V).

When the electrochemical performance of LTO electrodes with CMC, GG, TG binders were compared, they exhibited an initial reversible charge capacity of 169.3, 176.3 and 179.7 mA h g^−1^ respectively. Among them, GG offered the highest Columbic efficiency of 97.26 %, whereas CMC and TG offered 93.98 % and 93.44 %, respectively and, the CV data also supported this. Thus, GG was found to be a more active binder than TG and CMC since after 100 cycles, GG was able to deliver a revesible capacity of 160 mA h g^−1^, whereas TG and CMC delivered 150.1 and 147.5 mA h g^−1^respectively. The LTO@GG electrode exhibited higher capacity than the LTO@CMC as well as LTO@TG irrespective of the current rate. Although, the difference was higher at increased rate of charge‐discharge.

CMC, GG and TG are insulating polymers and, therefore, their critical role in LTO cannot be differentiated based on their electrical resistance. However, according to the 180° peeling experiments, linear CMC was found to possess higher adhesive capability than branched GG and TG. Ironically, cyclic performances of LTO@CMC, LTO@GG and LTO@TG were found to be the opposite of their adhesion capability. This was primairily due to the nearly zero volume change of LTO during charging/discharging cycle. In addition, X‐ray photoelectron spectroscopy (XPS) revealed that GG and TG were uniformly distributed on the surface of super P carbon.^[38]^ In the Ti 2p spectra, the highest peak intensity was observed for the LTO@GG electrode (Figure 8c). This could only be possible if the LTO electrode was directly exposed to the electrolyte. This direct exposure was possible because the surface of LTO was covered by GG in a uniform and narrow manner resulting from the weak H‐bonding between the backbones due to the high degree of branching. In addition, according to the morphological study, LTO@GG electrode has the best dispersion among them. Moreover, among these binders, GG uptook the highest amount of electrolyte (6.74 %) over a period of 48 h (Figure 8d). The electrolyte uptake experiment was carried out in the absence of solvent since the binder could also uptake solvent and, provide error in actual electrolyte uptake data. Thus, this electrolyte uplake was in large and appropriate to facilitate the movement of Li^+^ ions while keeping the morphology and crystalinity intact. This again could be due to the weak H‐bonding between the backbones resulting from its “garland of leaves” like conformation.^[106]^ Furthermore, the electrochemical impedance spectra (EIS) showed that LTO@GG has the highest charge transfer resistance (CTR) than LTO@TG and LTO@CMC. This correlated well with the fact that LTO@GG exhibited highest intensity in the Ti 2p spectrum and, uptook highest amount of electrolyte which faciliated rapid transfer of Li^+^ ions. Therefore, it was conferrred that GG was superior as a binder for LTO than TG and CMC.

Chemically modified GG, for example, hydroxypropyl GG (HPGG) was employed as binder for LTO negative electrode in conjunction with a intrincically conductive polymer (ICP), for example, poly‐3,4‐ethylenedioxythiophene/polystyrene sulfonate (PEDOT:PSS).^[114]^ The performance of LTO with the hybrid binder HPGG/PEDOT:PSS was compared with PVDF and CMC. It was found that upto 100 cycles, the specific capacity of LTO@HPGG/PEDOT:PSS electrode did not alter, however, corresponding LTO@PVDF/PEDOT:PSS showed a decrease which indicated a lower stability. Even after 200 cycles, the specific capacity loss of the LTO@HPGG/PEDOT:PSS electrode was only −1.8×10^−3^ mA h g^−1^.

### 2.4 Lithium Nickel Manganese Oxide Based Cathode

Although water soluble biopolymer binders were able to enhance the performance of the anode in LIBs, their application for lithium nickel manganese oxide (LiNi_0.5_Mn_1.5_O_4_, LNMO) cathode was not studied well. This was primarily because the high‐voltage LNMOs are highly senstive towards water and, corrosion of the aluminium current collector due to high pH (>11) as well as lithium leaching. Synthetic water soluble co‐polymers, for example, SBR was also found to be unstable at elevated potential and tend to oxidize. In an attempt to overcome aforementioned issues, Kuenzel et al. reported the use of GG as a binder for LNMO along with the utilization of appropriate processing additives, for example, phosphoric acid (PA) and, a crosslinking agent, for example, citric acid (CA).^[115]^ The cross‐linked CA‐GG exhibited a characteristic peak of the ester group at 1722 cm^−1^ which was in accordance with similar compounds.[^37^, ^81^, ^116^, ^117^] The crosllinked CP‐GG (CP=CA+PA) with 3 % binder loading exhibited increased long‐term cycle stability. Even, LNMO@GG was showing 92 % retention of capacity (107 mA h g^−1^) after 400 cycles and, this is much higher than corresponding LNMO@CMC. On the other hand, the LNMO@CP‐GG exhibited a slightly higher retention of capacity (110 mA h g^−1^, 96 %). Remarkably, the LNMO@CP‐GG was found to be stable above 120 mA h g^−1^ at relatively low charge/discharge rate (C/3). Enhanced coordination of the CP‐GG binder with the active material having oxides was responsible for the observed superior electrochemical performance of LNMO@CP‐GG over LNMO@CP‐CMC. There was no significant differences betweeen the LNMO@CP‐GG and LNMO@CP‐CMC when the C rates were low (C/2 to C/10). However, the LNMO@CP‐CMC performed better than LNMO@CP‐GG at elevated C rate. This has been attributed to the higher CTR of LNMO@CP‐GG because of the extended coordination of CP‐GG with the active electrode material. Remarkbaly, the cyclic stability of crosslinked GG and CMC (GG‐CP‐CMC) was slightly better than that of LNMO@CP‐GG as well as LNMO@CP‐CMC (Figure 8e). At 1 C, the LNMO@GG‐CP‐CMC (3 wt% binder) was able to provide a specific capacity higher than 120 mA h g^−1^ which was found to be the best. Interestingly, when the LNMO@CP‐GG was coupled with graphite@CMC anode, a capacity retention of 80 % along with superb cyclic stability was achieved after 1000 cycles. Loss of nickel or manganese was responsible for slight decrease of capacity and, GG has been thought to play a critical role by acting as a scavanging agent.[^118^, ^119^]

### 2.5 Lithium Nickel Manganese Cobalt Oxide Based Cathode

Development of lithium nickel manganese cobalt oxide (NMC) cathodes using aqueous fabrication methods did not progress much due to the similar reasons mentioned for LNMOs (section 2.4.). However, there are few promising examples, for example, NaCMC as a binder for NMC exhibited better rate stability than the corresponding PVDF binder. In addition, LiNi_0.5_Mn_1.5_O_4_ and LiNi_0.4_Mn_0.4_Co_0.2_O_2_ (NMC‐442) cathodes also exhibited superb cyclic stability with NaCMC than PVDF.[^120^, ^122^]

Recently, suitability of chemically modified GG namely HPGG and hydroxypropyltriammonium chloride GG (HPTACGG) as binders for NMC, for example, Li[Ni_0.33_Mn_0.33_Co_0.33_]O_2_ cathode has been evaluated.^[123]^ TGA experiments showed that there was slight weight loss of GG, HPGG, HPTCGG in between 50–100 °C and, all of them were found to be stable upto 200 °C. Above 200 °C, the stability of HPGG was found to be the highest followed by GG and HPTCGG and, ∼300 °C, drastic weight loss was observed for all of them due to the breakage of backbones. Above 4 V, only negligible amount of current flowed in the first cycle of CV experiment. Therefore, GG, HPGG, HPTCGG were thermally and electrochemically stable enough to be employed as binder for NMC.

**Figure 9:**
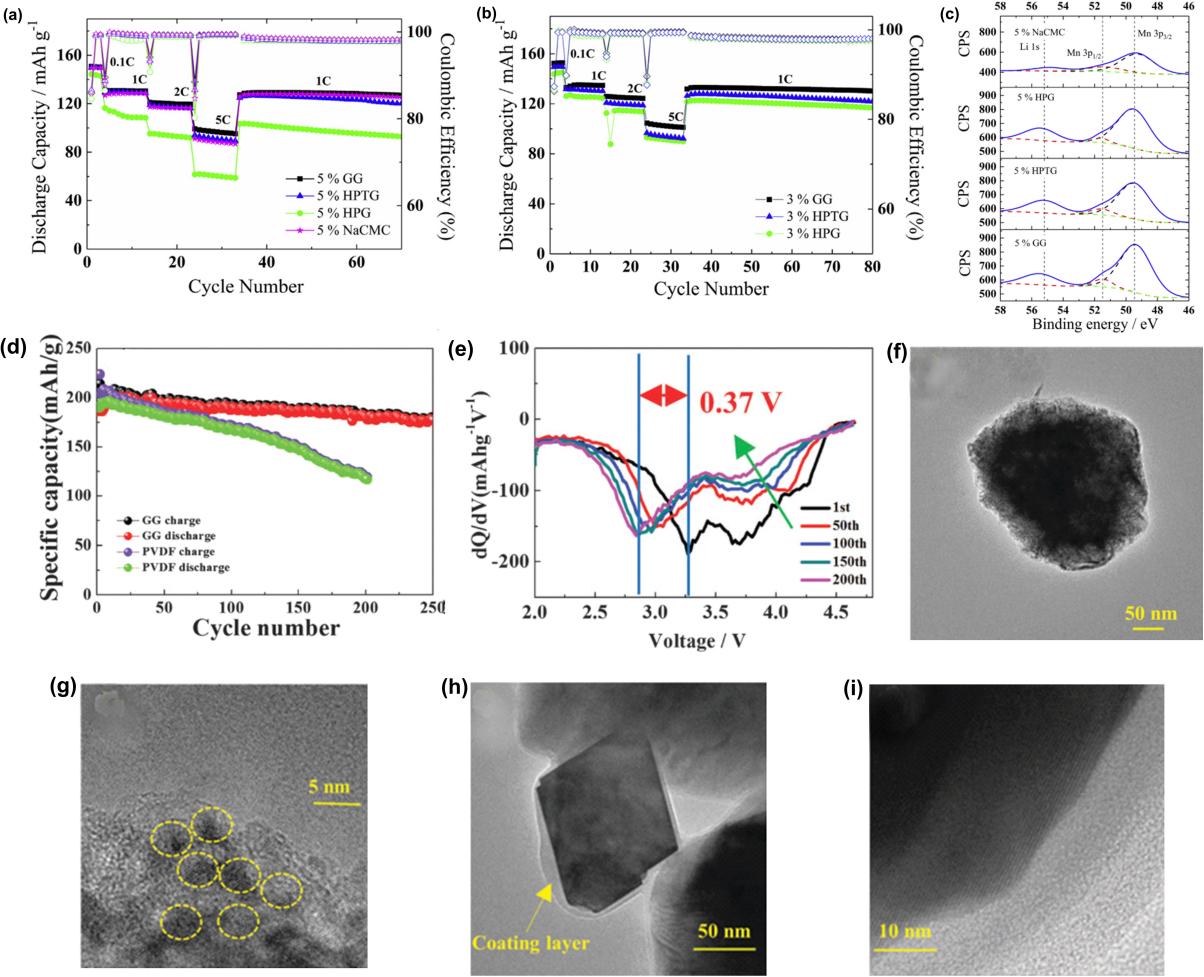
(a) Cyclic performance of NMC@GG, NMC@HPTGG, NMC@HPGG, NMC@NaCMC half cells with 5 % binder at 20 °C. (b) Cyclic performance of NMC@GG, NMC@HPTGG, NMC@HPGG half cells with 3 % binder at 20 °C. (c) XPS spectra of NMC@NaCMC, NMC@HPGG, NMC@HPTGG, NMC@GG with 5 % binder. Reproduced from Ref. [123], Copyright 2018, Elsevier. (d) Cyclic performance of LLRO@GG and LLRO@PVDF at 100 mA g^−**1**^. (e) dQ/dV plot for LLRO@GG cell. (f) TEM image of LLRO particles with PVDF (after 200 cycles). (g) HRTEM image of LLRO particles with PVDF (after 200 cycles). (h) TEM image of LLRO particles with GG (after 200 cycles). (i) HRTEM image of LLRO particles with GG (after 200 cycles). Reproduced from Ref. [129], Copyright 2016, Royal Society of Chemistry.

The electrochemical performance of NMC@GG, NMC@HPGG, NMC@HPTCGG, and NMC@NaCMC electrodes were comparable upto 2 C current rate (Figure 9a). On the other hand, NMC@GG exhibited better performance than others when the current rate was 5 C (Figure 9b). Notably, NMC@GG, NMC@HPGG, NMC@HPTCGG, and NMC@NaCMC exhibited >98 % Coulombic efficiency. The XPS spectra revealed that Li 1s peak intensity was lowest for NMC@NaCMC and, this could be due to a thicker coating layer compared to NMC@GG (Figure 9c). Surprizingly, the rate performance of NMC@HPGG was found to be lower than NMC@NaCMC despite the former had a thinner coating layer. This indicated that coating thicknesss was not the single factor which affected the rate performance. However, the amount of binder was found to affect the rate performance of all electrodes to a great extent. Higher amount of binder has lowered both specific capacity and capacity rentention of all electrodes. For example, capacity rentention and specific capacity of NMC@HPGG electrode at 80th cycle was improved from 77.5 % and 90 mA h g^−1^ to 92.5 % and 116.6 mA h g^−1^ respectively when the amount of HPGG was lowered from 5 % to 3 %. The XPS experiment revealed that solid electrolyte interface (SEI) formation on NMC was barely affected by the nature of the binders. On the other hand, thicker SEI containing LiF, Li_x_PF_y_, Li_x_PO_y_F_2_ salts, was formed on the graphite anode when NaCMC binder was used instead of GG. This indicated a lower chemical reactivity of GG than NaCMC at the graphite anode and possibly, exchange of Li^+^ ion with Na^+^ ion of NaCMC was reponsible for the observed difference.^[124]^

### 2.6 Layered Lithium‐Rich Oxide Based Cathode

Recently, the layered lithium‐rich oxides (LLROs) have emerged as a very promising material for cathode in LIBs. The LLROs have very high capacity (∼250 mA h g^−1^) and, they are nanocomposites of LiMO_2_ (M=Co, Ni, Mn, etc.) and Li_2_MnO_3_.[^125^, ^126^] However, the commercialization of LLROs has been halted due to some drawbacks, for example, (i) substantial amount of capacity loss happens because the Li_2_MnO_3_ component undergoes irreversible reaction during the first charge/discharge cycle, (ii) poor rate capability resulting from the lithium ion diffusion coefficient as well as low conductivity, (iii) capacity and voltage fade drastically during long charge/discharge cycle.[^127^, ^128^, ^129^, ^130^] Several approaches, for example, surface modification, crystal plane tuning and, introduction of spinel oxides have been taken to overcome aforementioned drawbacks. However, the drastic voltage fading issue continued to persist and, phase transformation has been thought to be the main reason. Structural modifications by changing synthetic routes have been studied but, they are not efficient and simple for large scale production.

In this regard, Zhang et al. reported that the voltage‐fading issue in LLROs can be suppressed by employing GG as a binder.^[133]^ It was observed that swelling of GG in electrolyte, for example, 1 M LiPF_6_ was minimum. In addition, GG was also dissolved very well in the electrolyte. After 100 cycles, the voltage and capacity fading of LLRO@PVDF was drastic, while for LLRO@GG it was minimal. More importantly, LLRO@GG was able to retain >90 % of capacity after 200 cycles while LLRO@PDVF retained only 62.4 %. After 100 cycles, LLRO@GG delivered a discharge capacity of ∼186 mA h g^−1^ while LLRO@PVDF delivered ∼162 mA h g^−1^. (Figure 9d) Interestingly, the average discharge voltage (ADV) of LLRO@GG after 200 cycles was 3.13 V which was 0.35 V less than the first ADV. On the other hand, the ADV of corresponding LLRO@PVDF after 200 cycles was 2.84 V which was 0.64 V less than the first ADV. This clearly showed that GG played a crucial role to suppress the voltage‐fading issue and enhanced the cycle performance of LLRO.

In the dQ/dV plots, both LLRO@PVDF and LLRO@GG exhibited discharge peaks at 3.7 V and 3.2 V which represents the Ni^+2^/Ni^4+^ redox system and reduction of Mn^4+^ to Mn^+3^. For the LLRO@PVDF, the peak at 3.7 V started to disappear with cycling and, the peak at 3.2 V gradually shifted to 2.62 V when 200 cycles were approaching. On the other hand, the peak at 3.7 V did not disappear for LLRO@GG and, the peak at 3.2 V shifted to only 2.88 V. This indicated that the Ni^+2^/Ni^4+^ redox system can be protected as well as the votage‐fading can be suppressed if GG is empolyed as a binder in LLRO. After 200 cycles, the transmission electron microscope (TEM) and high‐resolution transmission electron microscope (HRTEM) images of the disassembled electrode revealed that the surface of particles was coarse and, many islands were observed in which PVDF was used as a binder. This has been attributed to the acidic species arose from the electrolyte. However, particle surface was found to be smooth in case of GG and, this indicated that side reactions and corrosion of electrode was hindered via the stabilization of the Ni^+2^/Ni^4+^ region.

Table 2 summarizes the applications of GG and its derivatives in various LIBs. Comparison of GG as a binder with most commonly used binder PVDF as well as other biopolymer binders has also been shown. In all these examples, GG and its derivatives exhibited superior discharge capacity compared to other binders. It is also worthy to note that N‐GG‐XG exhibited superior discharge capacity than GG or XG alone. Most of these evaluations were carried out in small scale laboratory settings. Therefore, large scale evaluations are needed to further validate these findings.

**Table 2:** Summary and comparison of GG as a binder for LIBs.

Binder	Cell	Discharge Capacity [mA h g^−1^]	Coulombic Efficiency [%]	Reference
GG	SiNP/Li Foil	3364^[a]^	88.3^[a]^	[82]
SA	SiNP/Li Foil	2195^[a]^	82.5^[a]^	[82]
PVDF	SiNP/Li Foil	1232^[a]^	50^[a]^	[82]
GG	Li Foil^[b]^/S@pPAN	1375^[b]^	99.99^[b]^	[111]
CMC	Li Foil^[b]^/S@pPAN	1250^[b]^	–	[111]
PVDF	Li Foil^[b]^/S@pPAN	958^[b]^	–	[111]
GG	Li Wafer^[c]^/Sulfur	810^[c]^		[112]
XG	Li Wafer^[c]^/Sulfur	810^[c]^		[112]
N‐GG‐XG	Li Wafer^[c]^/Sulfur	913^[c]^	94	[112]
PDVF	Li Wafer^[c]^/Sulfur	636^[c]^	–	[112]
Gelattin	Li Wafer^[c]^/Sulfur	461^[c]^	–	[112]
GG	LTO/Li Foil	160^[d]^	97.26	[117]
TG	LTO/Li Foil	150.1^[d]^	93.34	[117]
CMC	LTO/Li Foil	147.5^[d]^	93.08	[117]
PEDOT:PSS/HPGG	LTO/Li Foil	142.5^[e]^	–	[118]
PEDOT:PSS/PVDF	LTO/Li Foil	137.5^[e]^	–	[118]
CP‐CMC	Graphite/LNMO	125^[f]^	90	[119]
GG−X‐CMC	Graphite/LNMO	119^[f]^	88	[119]
CP‐GG	Graphite/LNMO	119^[f]^	87	[119]
GG	Graphite/NMC	155^[g]^	>98	[127]
HPGG	Graphite/NMC	121^[g]^	>98	[127]
HPTCGG	Graphite/NMC	122^[g]^	>98	[127]
NaCMC	Graphite/NMC	116.7^[g]^	>98	[127]
GG	Li Foil/LLRO	186^[h]^	>98	[134]
PVDF	Li Foil/LLRO	170^[h]^	>98	[134]

[a] At a current density 2100 mA h g^−1^ and initial efficiency, counter/auxiliary electrode=lithium foil, SiNP loading=0.2–1.1 mg cm^−2^. [b] After 50 cycles at C/5, counter/auxiliary electrode=lithium foil (pure), Coulombic efficiency after 100 cycles at 7 C, S loading=0.78 mg cm^−2^. [c].After 60 cycles at 0.5 C, counter/auxiliary electrode=lithium wafer, S loading=0.78 mg cm^−2^. [d] After 100 cycles at 1 C, counter/auxiliary electrode=lithium foil, LTO loading=80 %. [e] After 100 cycles at 1 C. [f] After 100 cycles at 0.1 C. (g) After 80 cycles, Quasi‐reference electrode=Li metal, NMC and graphite loading=5.21–5.96 and 3.76–476 g cm^−2^ respectively. [h] After 100 cycles, LLRO loading=80 %.

## 3 GG as a Separator for LIBs

Separators are typically porous membranes and placed between the anode and cathode. In terms of safety issues, separators in LIBs play a critical role by enabling mobilty of Li^+^ ions, preventing contact between the anode and cathode. In addition, it helps to keep substantial amount of liquid electrolyte having ion‐conductive property. Change in the shape of separator during over‐heating and, formation of lithium dendrite can cause short circuit which can lead to fire and explosion of LIBs. Polyethylene (PE) and polypropylene (PP) or their combination are most commonly used separator for LIBs. Despite their excellent chemical stability and mechanical performance, they are thermally not so stable as they tend to melt at higher temperature. Furthermore, PP/PE based separtors exhibit poor affinity towards Li^+^ ion containing electrolytes. To address these issues, ceramic based separators have been developed using inorganic fillers, for example, Al_2_O_3_, SiO_2_.

Recently, a combination of HPGG and SiO_2_ (4 : 1 weight ratio) has been developed as a high temperature stable and low cost, environment benign separator for LIBs.^[79]^ The membrane was uniform and homogeneous probably due to the covalent bond formaion between silica and HPGG (HPGG‐OSi). This has been attributed to the hydrophobic nature of silica particles, higher porosity and chemical structure of GG which made the membrane more flexible. The membrane had a thickness of 30–50 μm and, suitable porosity (52 %) required for LIBs.^[131]^ On exposure to electrolyte, the membrane exhibited superior wettability as its weight was increased by 290 % and 370 % after 30 min and 60 min respectively.

According to the TGA experiment, the mebrane along with its component was stable upto 200 °C under N_2_ as well as O_2_ atmosphere. As a component of the membrane, HPGG exhibited stablity upto 240 °C under N_2_ than O_2_ atmosphere. At temperature above 240 °C, weight loss was observed for similar reasons described for LTO (Section 2.3.). The membrane was also found to be thermo‐mechanically stable since there was only 0.5 % weight loss in the isothermal test at 180 °C under N_2_. More importantly, the size of the menbrane did not alter when exposed to 180 °C for 12 h under N_2_. According to the linear sweep voltammetry (LSV) experiment, the membrane exhibited similar behavior like reference glass fiber separator. There was no current flow upto 4 V versus Li/Li^+^ and, the observed electrochemical stability window was upto 5 V versus Li/Li^+^. In practical test, the NMC/Li and LTO/Li half cells with HPGG‐SiO_2_ separator, were able to deliver discharge capacity of 151 mA h g^−1^and 161 mA h g^−1^ respectively. These indicated that at the cathode, full redox reaction had occured. Furthermore, no decomposition or anomalous reactions were observed on the separator. Therefore, the SiO_2_‐HPGG separator was found to be very promising and, could potentially be employed in full LIBs.

## 4 Summary and Outlook

In this minireview, the application of GG as a binder for electrodes and separator for LIBs have been discussed. The electrochemical performance of GG has been compared with PVDF, SA, CMC, LBG, XG and TG. Being an environmentally beign, non‐toxic and low cost heteropolysaccharide, GG has been found to be very promising as a binder for anodes in LIBs. GG possesses higher flexibility and tensile strength than CMC.[^132^, ^133^, ^134^, ^135^, ^136^] As a binder, GG was able to address several issues like volume changes during charging/discharging process, cyclic performance, rate capabilities of silicon‐based anodes. In addition, GG also provided thermal stability of anodes by maintaining structure and enhancing the mechanical strength of the electrode. Furthermore, GG was also able to reduce the “shuttle effect” in lithium‐sulfur batteries. Despite the initial setbacks, GG has also been found to be quite promsing as a binder for cathodes, for example, NMC. In addition, chemically modified GGs, for example, HPGG, HPTCGG have also been evaluated as binders for LIBs and, upto specific current rate their electrochemical performance was comparable to NaCMC. Moreover, combination of SiO_2_ and HPGG was also employed as a separator for LIBs and, found to be very promising.

In terms of electrochemical performance, no other batteries may ever surpass LIBs. However, the average cost to mine and refine a ton of lithium is ∼$15,000 and, this still makes it a challenge for the car industry to make HEVs and EVs afforable among population. In this regard it is worthy to mention that water soluble binders, for example, GG can provide several advantages. Naturally abundant water as a solvent for natural binders, is much cheaper than conventional solvent NMP (0.015 $/kg vs. 1—3 $/kg). Having a lower boiling point than NMP, water also can help to speed up drying process during fabrication of LIBs. In addition, solvent recovery step can also be avoided. All these advantages can potentially lower the manufacturing cost of LIBs.

The advancements discussed in the preceeding sections, were majorly carried out in laboratory settings where coin‐cell configuration was used. Therefore, it is necesary to test GG in large scale manufacturing process of advanced LIBs. In particular, testing in already commercialized LIBs may be easily adaptable and provide valuable insight.

These recent applications of GG and chemically modified GG as green binder and separator for advanced LIBs are encouraging for further modification of GG. In particular, multifunctional GG may potentially render additional benefits, for example, self‐healing, strong adhesion, excellent ionic conductivity, high elasticity and, this may further improve electrochemical performance of advanced LIBs. In this regard, GG and it derivatives may also find potential application as a green binder/separator in sodium ion battries (SIBs).[^137^, ^138^, ^139^, ^140^] We anticipate that this minireview will stimulate further research on GG and its derivatives as an abundant, low‐cost, renewable, and green binder/separator for next generation sustainable energy storage devices.

## Author Contributions

SS conceptualized the topic. SK wrote the first draft of the manuscript. SS reorganized and further modified the manuscript. All authors carried out proof‐reading of the manuscript. All authors approved the final version of the manuscript.

## Conflict of interest

The authors declare no conflict of interest.

5

## Biographical Information

*Simran Kaur completed B.Sc. Chemistry (Hons) degree from the Lovely Professional University, Phagwara, Punjab, India. She worked in the research group of Prof. Dr. Soumava Santra. She is currently pursuing her M.Sc. degree in industrial chemistry. Her research interests include organic synthesis, food science, industrial chemistry*.

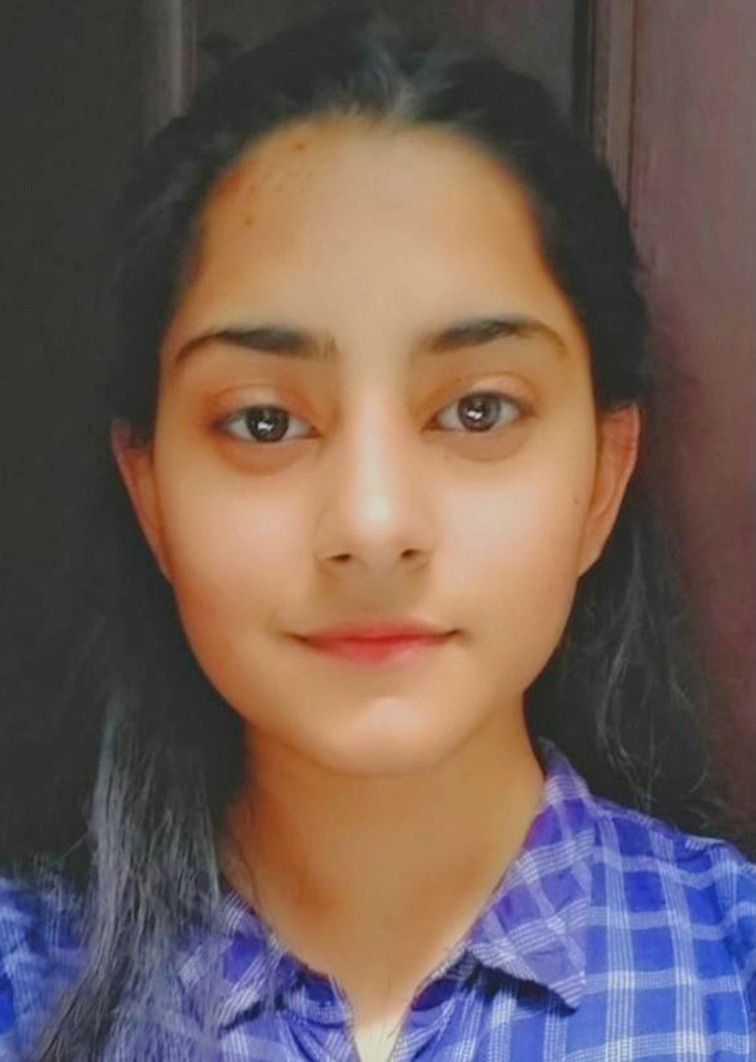

## Biographical Information

*Soumava Santra is an Assistant Professor at the Lovely Professional University since 2019. He completed his Ph.D. and post‐doctoral research at Wayne State University, USA, supervised by Prof. Peter R. Andreana and Prof. Aloke K. Dutta respectively. He further persued brief post‐doctoral research at the University of Windsor, Canada with Prof. H. Eicchorn and University of Toronto, Canada with Prof. Mark Nitz. His research interest includes development of synthetic methodology, green and sustainable chemistry, material chemistry, natural products, organic synthesis, small molecule inhibitors, chemical biology, drug design and drug discovery*.

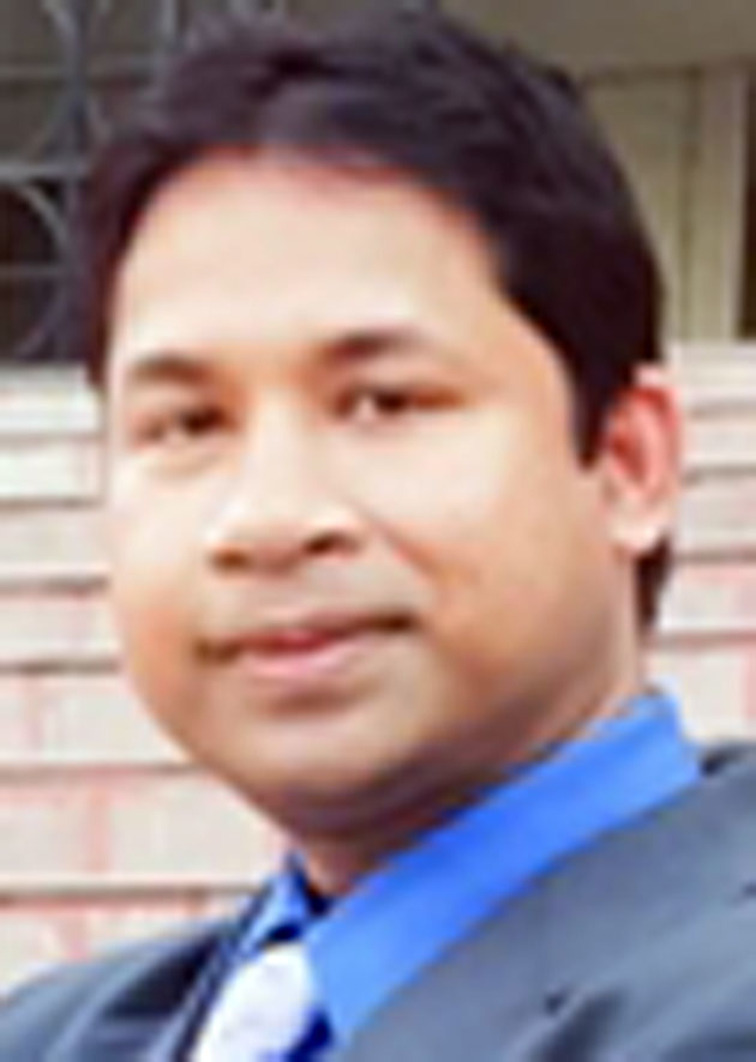

## Data Availability

Data sharing is not applicable to this article as no new data were created or analyzed in this study.
